# Field cancerization in the colon: a role for aberrant DNA methylation?

**DOI:** 10.1093/gastro/got039

**Published:** 2014-01-13

**Authors:** Yanxin Luo, Ming Yu, William M. Grady

**Affiliations:** ^1^Department of Colorectal Surgery, The Sixth Affiliated Hospital, Sun Yat-Sen University, Guangzhou, P.R. China, ^2^Gastrointestinal Institute, Sun Yat-Sen University, Guangzhou, P.R. China, ^3^Clinical Research Division, Fred Hutchinson Cancer Research Center, Seattle, WA, USA and ^4^Department of Medicine, University of Washington School of Medicine, Seattle, WA, USA

**Keywords:** colorectal cancer, field cancerization, epigenetic alterations, DNA methylation

## Abstract

Colorectal cancer is the third most common cancer worldwide and arises secondary to the progressive accumulation of genetic and epigenetic alterations in normal colon cells, which results in a polyp-to-cancer progression sequence. It is known that individuals with a personal history of colon adenomas or cancer are at increased risk for metachronous colon neoplasms. One explanation for this increased risk could be field cancerization, which is a phenomenon in which the histologically normal tissue in an organ is primed to undergo transformation. Epigenetic alterations appear to be promising markers for field cancerization. In this review, we discuss field cancerization in the colon and the data supporting the use of methylated DNA as a biomarker for this phenomenon.

## INTRODUCTION

It is commonly accepted that colorectal cancer (CRC) develops through a polyp-to-cancer progression sequence, in which normal colorectal epithelium transforms into an adenoma, which then progresses to cancer via the accumulation of progressive molecular changes, including both genetic and epigenetic alterations [1]. It is generally assumed that this sequential progression towards a tumor begins with a rate-limiting mutation in a critical growth-control gene (‘gate-keeper' gene, e.g. APC) that favors the clonal expansion of cells that acquire this mutation, and that the cells that carry gatekeeper mutations inevitably become adenomas as they acquire additional mutations [2]. However, recent studies have provided evidence that the road to CRC development can begin long before the appearance of the aberrant crypt focus, the first histologically recognizable alteration in the polyp-to-cancer sequence. Colon epithelial cells can acquire pro-tumorigenic mutations that are insufficient to cause morphological change, but which pre-dispose to subsequent tumor development. The clonal expansion of these mutant clonal populations can lead to the formation of large patches or ‘fields' of tissue that are primed to become neoplastic cells. This process has been appropriately termed ‘field cancerization’ [3].

The concept of field cancerization defects, or ‘field defects', was introduced by Dr. Slaughter in 1953 when studying oral squamous cell carcinoma [4]. The authors did not provide a clear definition of this phenomenon but the concept of field cancerization was proposed, based on the following findings: (i) the frequent concurrence of multiple independent tumors (*n* = 88/783 patients), which was much higher than expected by chance alone; and (ii) microscopic abnormalities with pre-cancerous changes, which were observed in grossly benign-appearing contiguous tissues. The authors suggested that field cancerization might be an important factor in the recurrence of oral cancer after resection, as well as in the multicentricity of oral cancers in people with no obvious predisposition to cancer (i.e. hereditary syndrome, etc.). Obviously, this concept is based on the assumption that the multiple tumors that arise from a ‘cancerized field' have developed independently, which does appear to be the case in the majority of the organ systems where it has been purported to occur, such as the bladder and the colon [5]. In this review, we summarize the current evidence that supports the occurrence of field cancerization defects in the colon and consider possible mechanisms for this phenomenon. We also discuss the potential for field cancerization markers to be used to identify individuals at increased risk for colorectal cancer and to be used to direct chemoprevention strategies.

## FIELD CANCERIZATION AND COLORECTAL CANCER

The possibility of field cancerization in the colon was first suggested by the increased occurrence of flat dysplasia and colorectal cancer in individuals with inflammatory bowel disease. Individuals who have had ulcerative colitis or Crohn’s colitis for more than 10 years have an increased risk of colorectal cancer, presumably because of the tumor-inducing effects of chronic inflammation [6]. The risk of colitis-associated cancer increases with the extent of the colon that is inflamed and the duration of the colitis [7]. Notably, colitis-associated cancers typically arise from flat mucosa that has become dysplastic, and multiple areas in the colon can simultaneously develop these dysplastic changes. Thus, it is clear that there is a field cancerization process in the setting of chronic colitis, and a variety of molecular mechanisms have been identified that may mediate this phenomenon [8]. Mutant *TP53* and aneuploidy are among the alterations shown to be present in the normal-appearing colonic mucosa of patients with colitis [9].

In the situation of sporadic colorectal cancer, the observation that individuals who have a personal history of colon adenomas or adenocarcinoma have an increased risk of forming metachronous adenomas, suggests that there may be a field defect driving this increased risk [10]. The molecular data supporting this possibility is not as robust as it is for colitis-associated cancer, but recent studies have identified an increased occurrence of chromosomal aberrations in the normal colon epithelium adjacent to colon cancer, as well as other changes, including aberrant DNA methylation, which support the concept that field defects may occur in the colon and increase the risk of colorectal cancer [5, 11].

### Epigenetic alterations: their role in colon cancer formation and potential role in field cancerization in the colon

The molecular changes that drive carcinogenesis in the colon include gene mutations and epigenetic alterations, which provide a growth advantage to the cells that acquire these alterations and lead to the clonal expansion of the altered cells. These alterations are believed to be one of the fundamental processes that drive cancer formation in the colon. Recent studies have suggested that the epigenetic alterations may be the earliest alterations in the polyp-to-cancer sequence and may precede dysplastic changes in the mucosa [12].

With regard to the epigenetic alterations observed in colorectal cancer, aberrant DNA methylation has been the most extensively studied. Modifications in DNA methylation related to the development of cancer include two fundamental changes: (i) hypermethylation of CpG islands in gene promoters—which can result in silencing of tumor suppressor genes—and (ii) hypomethylation of repetitive genetic elements, which may create a susceptibility to genomic instability [13]. The aberrant DNA hypermethylation observed in cancers affects CpG rich regions, called ‘CpG islands’, which are often found in the 5’ region of genes. The methylation of these CpG islands can result in transcriptional silencing, presumably through effects on transcription factor binding and changes in chromatin structure [2, 14]. The aberrant DNA methylation of CpG islands is common in many cancers, including CRC [15–17]. The process of aberrant gene methylation appears to begin early in the adenoma-carcinoma sequence, and probably affects genes that mediate both the initiation and the progression of CRC [18]. For these reasons, alterations in DNA methylation have the potential to be epigenetic biomarkers for the stratification of cancer risk or the detection of early cancers.

DNA methylation is of particular interest in cancer formation, in light of evidence that suggests that it may play a role in mediating a field cancerization process (also known as ‘field effect’ or ‘field defect’) that predisposes tissue to neoplastic transformation [19–22]. Field carcinogenesis—which has been observed to occur in various types of malignancies, including colon, lung, pancreas, esophagus, and prostate—is based on the premise that the molecular alterations present within a neoplastic lesion are also present diffusely within the organ and that these effects predispose normal cells to become dysplastic [23].

In the last decade, a number of studies have provided evidence that aberrant DNA methylation may be a marker of a field effect. These studies have assessed the methylation status of specific loci in normal colon mucosa and have demonstrated an association between increased DNA methylation of selected candidate cancer-related genes and the presence of a concurrent neoplastic lesion located elsewhere in the colon [19, 24–26]; for instance, methylation of five genes that have been used to identify CIMP colorectal cancers (*RUNX3*, *SOCS1*, *NEUROG1*, *CACNA1G*, and *IGF2*) has been found to be increased in the morphologically normal colon mucosa of individuals with advanced proximal sessile polyps, the precursor lesion to CIMP cancers [13]. The occurrence of the methylated genes in the normal colon of patients with sessile polyps is suggestive of field cancerization because of the known increased likelihood of individuals with sessile polyps developing metachronous polyps, when compared to people without a personal history of polyps.

In addition to the CIMP genes, there are a number of other specific methylated genes that have been found to be potential markers for field cancerization in the colon. Methylation of the O^6^-methylguanine-DNA methyltransferase (*MGMT*) gene promoter as well as of the *P14^ARF^* locus has been found in the normal-appearing colorectal mucosa adjacent to colorectal cancer [19, 27]. With regards to methylated *MGMT*, the normal-appearing mucosa located within 1 cm of an adjacent colorectal cancer was more likely to carry methylated *MGMT* than the mucosa 10 cm away from the cancer, which suggests that the field defect is localized close to the tumor, although other studies have shown that the field is larger than this.

Another locus implicated as a marker of field cancerization in the colon is *EVL/miR-342*. Grady *et al.* found that *miR-342* and its host gene *EVL* were frequently methylated in colon adenomas and adenocarcinomas. They found that the normal colon mucosa 10 cm away from the colorectal cancer had methylated *EVL/miR-342* in almost half of the cases, whereas only 12% of normal colon mucosa from individuals without cancer had methylated *EVL/miR-342* detected [28].

Others have demonstrated a direct correlation between aberrant methylation of *APC*, *DKKI*, *CDKN2A/p16*, and *SFRP4* in the apparently normal colon mucosa of cancer patients and, to a lesser extent, of polyp patients [29]. Methylation of a panel of genes isolated from normal rectal biopsies from 113 subjects was able to discriminate between those with and without an adenoma present at the time of biopsy [30].

Another example of data supporting the role of methylated genes as field cancerization markers comes from a study by Belshaw *et al.*, who investigated the patterns of DNA methylation in 260 individual colonic crypts obtained from eight female patients with no evidence of colorectal disease and five with colorectal cancer. They found that the differential methylation of genes (*DKK1, WIF1, SFRP1, SFRP2* and *SFRP5*) associated with the Wnt signaling pathway was present in individual, morphologically normal crypts, which may contribute to the generation of a colonic field defect and to the stepwise development of colorectal neoplasia [26].

In light of the known occurrence of hypomethylated repetitive elements in colorectal cancer, hypomethylation of LINE-1, SAT-alpha, and SINE elements has also been assessed as a possible field cancerization marker. Kamiyama *et al.* found a correlation between hypomethylation of long, interspersed nucleotide element-1 (LINE-1) and an increased risk for multiple colorectal cancers [31, 32]. However, despite these findings, the role of LINE-1 hypomethylation is controversial as the results of a number of studies on this subject have yielded mixed results with respect to an association between LINE-1 hypomethylation and colon neoplasms [33, 34].

### Potential mechanisms for field cancerization

The concept of field cancerization was originally hypothesized to explain observations in relation to oral cancer [4], and is now proposed to occur in a variety of organs, including the lung [35], breast [36], esophagus [37], stomach [38], and colon [19]. However, the underlying molecular mechanisms responsible for field cancerization are still largely unknown, except for inflammation-associated field cancerization [39].

One potential mechanism that may generate a field effect involves the occurrence of somatic mutations or epigenetic alterations in stem cells and the clonal expansion of these histologically normal-appearing cells. A genetic explanation for field cancerization in the colon is rooted in our understanding that cancer is fundamentally a consequence of epigenetic and genetic alterations in cells [40]. In the colon, all epithelial lineages are derived from stem cells residing in the base of the crypt. Field cancerization could initiate from a stem cell that acquires a genetic or epigenetic alteration, which induces proliferation and expansion of the stem cell-like population. This expanding clonal population can form a patch of cells with the same genetic or epigenetic alterations. By virtue of having a growth advantage over its neighboring cells, this patch of cells can gradually expand into a field and displace the surrounding cells that lack the mutation. In the process of expansion, the sub-population of the cells within a patch may acquire additional genetic mutations and eventually evolve into a neoplasm [9, 41]. Although the data for the exact size of clonal patches in the colon is limited, field defects in the colon mucosa have been shown to involve patches measuring anywhere from 2 mm to >10 cm in diameter [9, 11, 19, 42].

Another potential mechanism is related to dietary exposures that may influence the epigenetic state of tissues such as the colon mucosa. There is some evidence that dietary folate and vitamin B exposure may affect the methylation state of the normal colon mucosa and create a state of predisposition to cancer [43, 44].

## CONCLUSIONS

There is a substantial body of evidence that field cancerization can occur in the colon and that it may influence the risk of developing primary and metachronous colon adenomas and colon cancer. Although the concept of field cancerization was proposed almost 60 years ago, our understanding of many fundamental aspects of this phenomenon is still rudimentary. Aberrant DNA methylation has been identified as a potential biomarker for field cancerization defects in the colon. The role that epigenetic alterations play in field cancerization is still under investigation. Despite our limited understanding of field cancerization, methylated genes appear to be promising biomarkers for cancer field defects and may be clinically useful in the future.

## FUNDING

This manuscript was supported by National Institutes of Health (NIH) National Cancer Institute (NCI) NIH awards RO1CA115513, P30CA15704, UO1CA152756, U54CA143862, and P01CA077852 (WMG); Burroughs Wellcome Fund Translational Research Award for Clinician Scientist (WMG).

**Conflict of interest:** None declared.
